# Retraction Note to: SOX2 Regulates lncRNA CCAT1/MicroRNA-185-3p/FOXP3 Axis to Affect the Proliferation and Self-Renewal of Cervical Cancer Stem Cells

**DOI:** 10.1186/s11671-022-03679-3

**Published:** 2022-03-30

**Authors:** Li Zhang, Chunjie Guo, Tiefeng Ji, Xin Chen

**Affiliations:** Department of Radiology, The First Hospital of Jilin University, Xinmin St. 71, Changchun, 130021 Jilin People’s Republic of China

## Retraction Note to: Nanoscale Res Lett (2021) 16:2 10.1186/s11671-020-03449-z

The Editors-in-Chief have retracted this paper because the conclusions are not reliable. After publication, the authors contacted the journal to state that they were unable to reproduce the results of the CHiP experiment in HeLa or other cell lines. Additional concerns were raised about some of the figures presented here but the authors failed to provide the original data. The Editors-in-Chief therefore no longer have confidence in the integrity of the data in this article.

The authors have not responded to any correspondence from the editor/publisher about this retraction.

